# Carotid Artery Plaque Identification and Display System (MRI-CAPIDS) Using Opensource Tools

**DOI:** 10.3390/diagnostics10121111

**Published:** 2020-12-21

**Authors:** Felipe P. Vista, Minh Tri Ngo, Seung Bin Cho, Hyo Sung Kwak, Kil To Chong

**Affiliations:** 1 Advanced Electronic Research Information Center, Jeonbuk National University, Jeonju 54896, Korea; boduke@jbnu.ac.kr; 2 Department of Radiology and Research Institute of Clinical Medicine of Jeonbuk National University, Biomedical Research Institute of Jeonbuk National University Hospital, Jeonju 54907, Korea; ngominu@gmail.com; 3 Electronic Engineering Department, Jeonbuk National University, Jeonju 54896, Korea; seunbgin1647@jbnu.ac.kr

**Keywords:** magnetic resonance imaging (MRI), carotid artery plaque, lumen and wall, digital imaging and communications in medicine (DICOM), open-source

## Abstract

Magnetic resonance imaging (MRI) represents one modality in atherosclerosis risk assessment, by permitting the classification of carotid plaques into either high- or low-risk lesions. Although MRI is generally used for observing the impact of atherosclerosis on vessel lumens, it can also show both the size and composition of itself, as well as plaque information, thereby providing information beyond that of simple stenosis. Software systems are a valuable aid in carotid artery stenosis assessment wherein commercial software is readily available but is not accessible to all practitioners because of its often high cost. This study focuses on the development of a software system designed entirely for registration, marking, and 3D visualization of the wall and lumen, using freely available open-source tools and libraries. It was designed to be free from “feature bloat” and avoid “feature-creep.” The image loading and display module of the modified QDCM library was improved by a minimum of 10,000%. A Bezier function was used in order to smoothen the curve of the polygon (referring to the shape formed by the marked points) by interpolating additional points between the marked points. This smoother curve led to a smoother 3D view of the lumen and wall.

## 1. Introduction

Cardiovascular diseases are one of the leading causes of death in the West [1]. Strokes and heart attacks are mostly caused by rupture of atherosclerotic lesion, resulting in blocking of distal vessels due to the release of emboli or the local formation of a thrombus [2]. The structural features that tend to cause lesions to rupture can be analyzed through magnetic resonance imaging (MRI), especially for large-sized vessels such as the carotid arteries [3,4]. Quantitative characterization of lesion distribution, composition, and size can be obtained from magnetic resonance (MR) images which can assist researchers in understanding lesion progression, pharmacologists in assessing the response of patients to drug therapy, as well as clinicians in evaluating the specific risks associated with a particular lesion [5,6]. These analyses can be facilitated by software tools that can make the work both simpler and more efficient.

Diabetes mellitus (DM) is known to be associated with a specific pattern of vascular change [7] wherein type 2 DM signifies a known risk factor for atherosclerosis but its specific influence on plaque vulnerability was not fully understood until Esposito et al. [8] explored this aspect. They investigated if MRI-based plaque imaging can be utilized to detect probable differences between diabetic and nondiabetic patients in terms of carotid plaque features. They also studied if MRI-detected high-risk lesion types can help assess if diabetic patients are at higher risk than nondiabetics in terms of cerebral ischemia development after endarterectomy of carotid artery stenosis. Plaque imaging through MRI represents one modality in atherosclerosis risk assessment. It permits the classification of carotid plaques as either high- or low-risk lesions (type I–type VIII). 

Cai et al. [8] introduced the modified American Heart Association (AHA) classification of Stary [9,10] specifically for MRI use (Table 1) which categorized the intermediate to advanced atherosclerotic lesions in the carotid artery and enabled the noninvasive identification of advanced lesions from early and intermediate plaques. Esposito gathered that lesion types IV to VI are high-risk unstable plaques that are more prone to rupture and lead to cerebral ischemia, as well as were certainly more dominant in symptomatic carotid artery stenosis patients compared to asymptomatic ones. They also showed that Type 2 diabetes mellitus can be a predictor for the development of carotid artery plaques, irrespective of the degree of stenosis or other risk factors. MRI provides a useful method for assessing the risk status of diabetic patients because it can detect differences in carotid plaque features when comparing diabetic with non-diabetic patients. Although MRI is generally used for observing the impact of atherosclerosis on vessel lumens, it can also show both the size and composition of plaque itself, as well as plaque information, thereby providing information beyond that of simple stenosis [11].

A software package for analyzing atherosclerotic arterial lesions visualized in vivo via MRI was initially presented by Kerwin et al. [1]. Known as quantitative vascular analysis system (QVAS), it allows the interactive identification of lesion and vessel boundaries, tissue class segmentation within lesions, quantitative analysis of lesion features, and three-dimensional rendering of lesion structure. A more advanced computer-based system for cardiovascular disease evaluation (CASCADE) has also been proposed by Kerwin et al. [12] to streamline and automate the analysis of carotid-artery MRI for evaluating atherosclerotic plaque burden. The present study focuses on the design and development of a software system for marking both the wall and lumen and then generating a 3D visualization, using freely available open-source tools and libraries. It was designed with the guiding principle that it should be a specific software tool free of feature bloat and avoiding feature-creep. The image loading/display of QDCM library was improved while the generated 3D view was smoother because of the use of quadratic Bezier function. The specific technical advantages/contributions of the proposed system are as follows:

- The loading of the images is not dependent on the specific names of the folders for each DICOM image type as long as each of the five folders contain valid DICOM files. All the labels/information displayed for each sub-window are extracted from the DICOM files themselves.
- Minimum 10,000% factor improvement in QImage loading for all the MRI image types in the modified code compared to the routine in the existing original QDCM library.
- In terms of system development, all the sub-windows for each of the MRI image type are inherited from a parent sub-window which makes it easier for the developer to write specific functions for the MRI images.

This paper is arranged with a brief backgrounder in the succeeding sub-section followed by a description of the proposed architecture in Section 2. The opensource tools utilized are given in Section 3 while the design and development process are discussed in Section 4. The resulting system is then presented in Section 5 followed by the concluding remarks.

### Background

Commandeur et al. [13] have reviewed recent advances in computed topography (CT) software/hardware technologies as well as machine learning algorithms for cardiovascular imaging that have led to an expansion of the clinical utility of CT. They elaborate on significant developments in CT hardware, such as faster gantry rotation that enables improved temporal resolution. CT has also enabled higher coverage of the patient and enhanced spatial resolution, resulting in faster acquisition. In particular, Cardiac CT software has been developed for characterizing coronary plaque and adipose tissue around the heart as well as measurement of non-invasive FFR. Machine-learning algorithms have improved detection and prognosis of risks of lesion-specific ischemia. Such improvements can be expected to continue, as they are being utilized in clinical procedures for image acquisition and analysis as well as predicting patient outcomes. It is worth noting that Coronary CT Angiography (CTA) has enabled the evaluation of atherosclerotic plaques that were previously measurable only via invasive procedures [14,15].

The advantages of MRI over CT or digital subtraction angiography (DSA) as a diagnostic tool for cerebrovascular pathology include excellent tissue contrast, blood-vessel visualization, as well as the use of radiofrequency pulses and magnetic fields instead of ionizing radiation. Harteveld et al. [16] have identified three assessment levels of cerebrovascular diseases using MRI: (a) pipes; (b) perfusion; and (c) parenchyma. Pipes refer to the arteries that feed the brain from the heart and the aortic arch, up to the carotid and vertebral arteries, the circle of Willis, as well as smaller intra-cranial arterial branches. Perfusion involves the volume of blood reaching brain-tissue level, including the vascular reserve and perfusion territories. Parenchyma is concerned with chronic and acute problems of brain-tissue damage. These include smaller microinfarcts, larger infarcts, and small vessel diseases such as lacunar infarcts, matter lesions, and microbleeds.

With regard to pipes, there have been numerous developments over the past decade related to 7 tesla (7-T) magnetic resonance imaging of the extracranial vasculature that feeds the brain. The focus of this research can be generally subdivided into vessel-wall and vessel-lumen imaging. Vessel-lumen imaging for cerebrovascular diseases is generally performed either through time-of-flight MR angiography (TOF-MRA) or phase-contrast MR angiography (PC-MRA). Ultrahigh-field MRI such as 7-T has a longer T1 relaxation time, and thus produces greater contrast between blood flow and surrounding tissues [17,18,19,20]. However, 7-T is less useful as a “one-stop-shop” imaging tool as well as especially challenging in performing robust imaging of the neck region [21]. Studies related to imaging of extracranial atherosclerotic plaque and vessel wall at 1.5T have received considerable attention over the past decade [22], with much of this attention focusing on characterizing proximal internal carotid artery atherosclerotic plaques as a possible method for identifying high-risk patients for carotid surgery (carotid endarterectomy).

Hosseini et al. [23] have shown that MRI-defined carotid-plaque hemorrhage (MRIPH) can independently predict recurrent ipsilateral ischemic events as well as stroke in symptomatic carotid disease, which can be useful in identifying patients for carotid intervention in lower risk-benefit scenarios. Singh et al. [24] have pointed out the effectiveness of clinically adopting carotid MRI in cardiovascular risk assessment, specifically as a potential imaging biomarker for future risk, due to its reliability and high level of accuracy. On the other hand, intracranial vasculature imaging is better performed using ultrahigh field MRI since intracranial arteries are smaller. Detailed imaging with higher spatial resolution is readily available at ultrahigh field strength within reasonable scan times. Works in this field can also be split into vessel wall and vessel lumen. 7-T may be seen as bridging the gap in vascular pathology between the larger extracranial and smaller intracranial arteries but there are distinct safety issues related to its use. Hoff [25] discussed these safety issues of great concern such as the increased forces on metallic implants, unpredictable tissue heating that arises at 7-T due to the radiofrequency as well as several bioeffects like magnetophosphenes, nystagmus and vertigo which are also a problem and more prevalent for 7-T as compared to lower-field strengths. Hansson et al. [26] recently presented their result on a large-scale three-year study on the effects experienced in 7-T MRI systems, specifically peripheral nerve stimulation (PNI) and caregiving wherein 63% of the subjects agreed that the experience was comfortable enough and a majority (93%) were willing to undergo 7-T MRI in the future either as patient while 82% were willing to do it for research purposes.

Ultrasound also performs well when utilized for carotid arterial plaque assessment. Roy Cardinal et al. [27] conducted a study wherein ultrasound noninvasive vascular elastography (NIVE) provided an understanding of in vivo mechanical imaging. Neovascularized and vulnerable plaques were detected based on shear and lower axial strains, lateral and axial translations, as well as higher cumulated axial strain to cumulated axial translation ratio. The American Society of Echocardiography through Johri’s [28] work reviewed the use of both two-dimensional (2D) and three-dimensional (3D) ultrasound for carotid arterial plaque quantification. They recommended standards for acquisition and measurement by formulating cardiovascular disease stratification.

Biermann et al. [29] have evaluated the impact of advanced software assistance on carotid artery stenosis assessment, specifically regarding inter-observer variance in users with varying levels of experience. Patients with suspected carotid-artery stenosis underwent dual-energy CT angiography on the head and neck. The images were blind-interpreted in the usual way by four readers with varying levels of expertise. An advanced vessel-analysis software tool was then used by the readers several days later for quantification such as automatic hard plaque and bone removal, vessel segmentation, or curved planar reformation creation. The results of the study showed that whereas the two more experienced readers achieved inter-observer variability results of very good (k = 0.85) and good (k = 0.78) respectively, the results for the less-experienced readers were only moderate (k = 0.6) and fair (k = 0.24). By contrast, the inter-observer variability results obtained when the readers were using the advanced vessel-analysis software were all in the good range (k = 0.77, k = 0.72, k = 0.71, and k = 0.77). The study thus demonstrated that standard image interpretation coupled with advanced software for vessel analysis produced very good results for experienced readers performing vascular lesion diagnosis. Even inexperienced readers were able to achieve good results during stenosis quantification when using advanced vessel-analysis software.

## 2. Proposed Architecture

### 2.1. Research Purpose

Analysis and diagnosis of plaque build-up in carotid arteries using MRI usually involve a manual process and take a long time, while also being harder for patients to visualize. A large number of software packages are commercially available but they are neither cheap nor affordable [12,30,31]. While open-source medical software systems with similar functionalities are available, they are complex and are often bloated with extra features unnecessary for the specific task at hand [32,33,34]. There is thus a need for a specific system tool of minimal cost that can provide effective assistance in processing carotid artery MRI images of patients, to expedite analysis and diagnosis as well as enabling patients themselves to gain a better understanding of their medical condition. While a number of open-source software packages are available, they are somewhat complicated to use. Accordingly, we set out to design and develop a single-purpose software tool for 3D visualization of marked lumen and wall that is as simple to use as possible.

### 2.2. Algorithm

The existing process for identifying carotid artery plaque usually involves loading and opening MRI image files and then displaying them to patients while explaining the findings. A major drawback of this method, however, is that it is left to the medical practitioner as to how to characterize the plaque build-up to the patient. For their part, patients are expected to visualize or imagine what the practitioner is referring to. The current manual method is time-consuming (approximately 30 min per patient). Shorter processing times mean that more cases can be diagnosed, as well as enabling practitioners to show patients 3D visualizations of plaque buildup.

**Figure 1:**
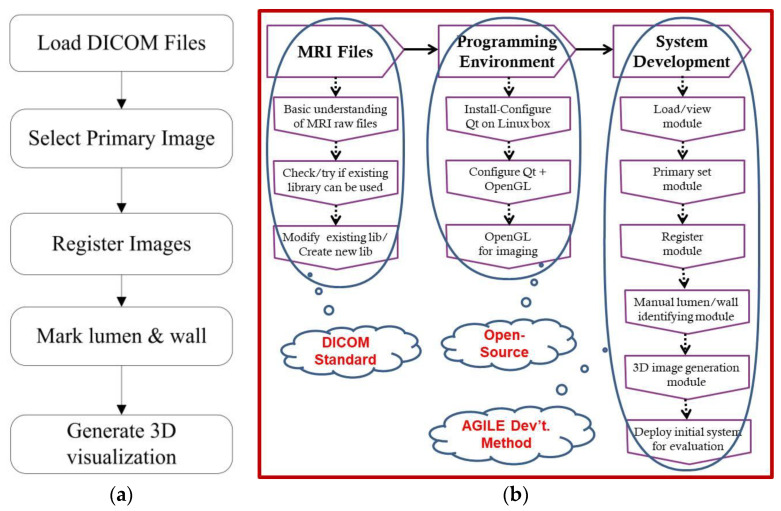
System: (**a**) simplified algorithm and (**b**) workflow.

A simplified version of the algorithm used in the proposed system is provided in Figure 1a, from loading of digital imaging and communications in medicine (DICOM) files to 3D visualization. The workflow (Figure 1b) is divided into three categories: (1) processing of MRI DICOM files; (2) settings and configuration for the programming environment, operating systems, as well as libraries to be used; and (3) design, development, and testing of the system itself.

## 3. Open Source Tools

### 3.1. Qt-Anywhere on Ubuntu Linux

Our research focused on utilizing Open Source tools as much as possible. We began by selecting the programming and development environment, followed by the existing library for processing DICOM files as well as for 3D-image rendering. The system we have designed and developed was based on the RBCDWBPA-SFA (Rapid By-Customer Demand with Business Process Approach with Systems Features Analysis) method on a desktop computer running a 64-bit Ubuntu operating system, using the Qt-anywhere 5.12.3 open-source version [35]. Qt-anywhere was selected as the programming environment because it is platform-independent in terms of design, development, and deployment. Although developed in an Ubuntu Linux environment, the program can thus be ported to a Microsoft Windows system with some minimal changes in the library. An advantageous aspect of Qt is its SIGNAL-SLOT feature, which allows data/information to be transmitted via signals to other parts of the system and to be processed by the slots. The SIGNAL can be seen as like a switch that triggers the execution of a function (the SLOT) as well as provide the necessary input for the slot to run properly.

### 3.2. DICOM and QDCM Library

DICOM [36] is the international standard for the interoperability of processes involving the transmission, storage, retrieval, printing, processing, and display of medical imaging data. It is very useful in meeting the diverse requirements of the various actors involved in medical imaging, including physicians (better access to images and reports assists in faster diagnosis), patients (faster and more effective care), as well as device manufacturers and medical institutions (by ensuring compatibility with other imaging devices). DICOM also plays an important role in the development of software systems related to medical imaging. The standard is managed by the Medical Imaging and Technology Alliance, which is a division of the National Electrical Manufacturers Association.

**Figure 2:**
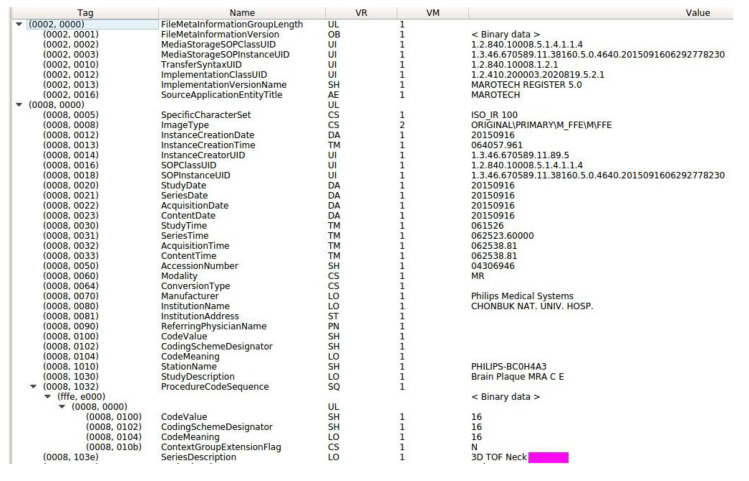
Extracted tag data.

DICOM information is grouped into datasets, which generally comprise four types of information: image data, whether raw or compressed; patient data (identification and demographics); technical information regarding the imaging device used; and exam, series and slice/image data. A DICOM data object comprises several attributes, including name, identification, and other details, as well as special attribute that contain image pixel data. One of these attributes, DICOM Modality, identifies the DICOM file type. Additionally, each of the other attributes includes a value multiplicity that tracks the number of data elements enclosed in the attribute. The primary components of a DICOM attribute are:

- A Tag (Figure 2): identifies the attribute; usually in (XXXX,XXXX) hexadecimal format; can be further split into group number and element number.
- A Name: descriptive text that describes the tag.
- A Value Representation (VR): identifies the data type and format of the attribute value.
- A Value Multiplicity (VM): defines whether an attribute can or cannot include multiple elements.
- The Value: the attribute itself.

QDCM [37] is a Qt-based open-source library that can be used for reading, writing, and modifying DICOM data. A DICOM data set is identified through its key, which is composed of two 16-bit values: a group number and an element number. Since DICOM tags are identified through this group and element key combination, the QDCM library maintains a dictionary of tag keys mapped to tag names and information tag data types.

### 3.3. OpenGL

OpenGL (Open Graphics Library) is the most widely used 3D and 2D graphics API (application program interface) in the industry, and is utilized on wide range of computer platforms [38]. It is both operating system and window system independent that is also network transparent. It runs on all major operating systems, including Windows (95/98, 2000, NT, 8, 10), MAC (OS, OS/2), almost all flavors of Linux (Ubuntu, Fedora, RedHat), BeOS, and OPENStep. OpenGL can be called using various languages, including Ada, C/C++, Fortran, Perl, Python, and Java, as well as offering complete independence from network topologies and protocols. OpenGL encourages innovation and accelerates application design and development via a comprehensive set of texture mapping, rendering, effects, and many other visualization tools. For developers, OpenGL’s advantages include being of industry standard, stability, reliability and portability, continuous development, scalability, ease of use, and extensive documentation.

## 4. Design and Development

**Figure 3:**
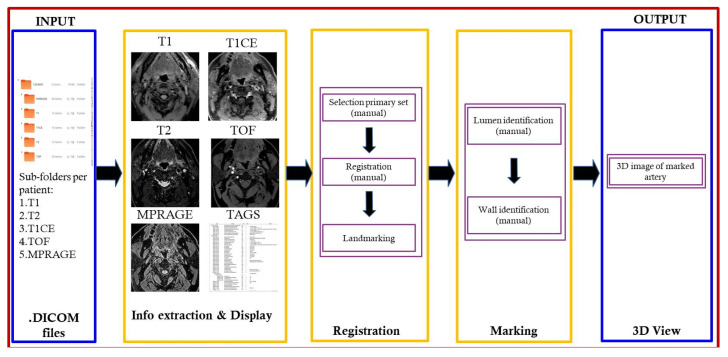
System design.

The current manual process includes the following major sub-processes: selection of primary image set, registration/landmarking of images, and marking/identification of the lumen/wall. The existing process was analyzed and reformatted into an algorithm of the proposed system, divided into the system design modules shown in Figure 3. It begins with loading and opening the MRI image files to display a 3D visualization of the carotid artery plaque. The step-by-step process is divided as follows:

1. Browse, open, and load MRI files from patient folder.
2. Select MRI image file to be set as the primary image set (T1, T2, T1CE, TOF, MPRAGE).
3. Rationalize the image set by ensuring that all image types are slice aligned and include the same number of images per set for processing.
4. Identify by marking lumen and wall for each image slice.
5. Generate 3D rendering of marked lumen and wall.

### 4.1. Image/Data Loading Module

This module begins by browsing for the patient directory folder and checking that it contains six sub-folders: one each for the T1, T2, T1CE, TOF, and MPRAGE, sorted alphabetically. After verifying that the sub-directories are not empty, the individual files are sorted, counted, and then loaded to display the extracted images, as well as patient information from the relevant DICOM files using the QDCM library. There is a separate sub-window for each of the MRI image types. 

A scrolling function between the loaded images involves monitoring the mouse scroll action in either an upward or downward direction. When a mouse scroll-up action is detected, the next image counter is checked as being either less than or equal to the total number of files in the subdirectory, while a mouse scroll-down checks that the current image counter is greater than or equal to zero (since the count starts from zero). The current image/information is then deleted and the image/information from the next or previous DICOM file in the sub-directory is processed.

#### Faster Image Loading

**Figure 4:**
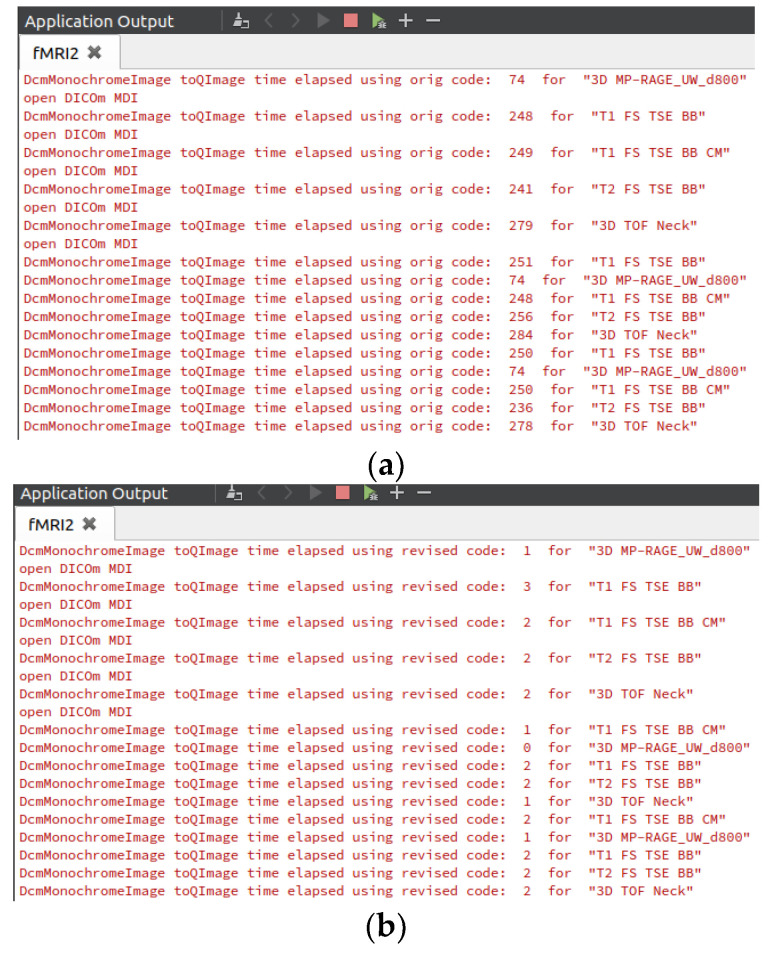
Improved image conversion process. (**a**) Original algorithm; (**b**) modified algorithm.

An important aspect in the development of this module was modifying the existing codes in the QDCM library to improve the time required to convert the image to QImage format for utilization in QT by utilizing registers and pointers for faster operation. Figure 4a,b with Table 2 show average conversion times for initial image loading, scroll up, and scroll down, as well as the improvement factor for the same set of DICOM files as the updates made to the existing algorithm.

### 4.2. Registration Module

This module is concerned with selection of the primary image set process and then finalizing the image sets to be utilized for marking. The primary image serves as the main image on which markup is performed, which is then automatically reproduced on the other (secondary) image sets. In primary image selection mode, the user can easily select the primary image set by hovering the cursor over the various sub-windows, clicking to select, then confirming the selection.

After the primary image set has been selected, the registration process can be activated. It begins with the selection of the initial or number 1 image in the other image sets, based on slice location value and the expertise of the user. The image sets can then be registered only when the initial image of all the secondary image types has been assigned. The main registration process checks that: (1) The primary and the secondary sets include the same number of images; (2) the “gap” or interval between successive images in the set is similar; and (3) all the image sets are aligned as closely as possible. 

The first step is required because T1 FS TSE BB, T1 FS TSE BB XM, and T2 FS TSE BB include the same number of DICOM files, whereas 3D MP-RAGE and 3D TOF usually include a much larger number. For the second step, the gap or interval between the images also varies depending on the image sets, with T1, T1 CE, and T2 usually including a gap of more than 1.8 while for 3D MP-RAGE and 3D TOF it is less than 1.8. Through these “Slice Location” values, all the image sets can automatically be set to have the same gaps or intervals, by alternately removing images from 3D MP-RAGE and 3d TOF. At the same time, the image slices are aligned based on the selected primary image for each image set. 

### 4.3. Marking Module

This module performs two functions: (1) marking the lumen, and (2) marking the wall. The marking procedure is performed by adding points by continuously moving the mouse clockwise around the selected lumen and then completing the point marking by double-clicking. Points already marked may have additional points added, while points may also be moved or deleted. This procedure is performed on the primary image and is reproduced on the secondary image sets.

The procedure for marking the wall can also be performed using a different color set for points and lines to distinguish between the two. The points essentially form a closed polygon for each image layer, each of which represents the marked lumen and wall. Each point set is saved as part of the node of a doubly linked list that contains pointers to the previous or next nodes, as well as the slice location value. It is worth noting that the marking process involves only the {X, Y} axis while the points in the polygon saved to the doubly linked list are in {X, Y, Z} format. This is made possible by deriving the Z-axis values from the slice location values for each image layer in the set. For the moment, the marking procedure described is performed on all images in the image set and is reproduced in all the image sets before the algorithm moves on to the next module.

### 4.4. 3D View Module

#### 4.4.1. Smoother Contour of Polygon

**Figure 5:**
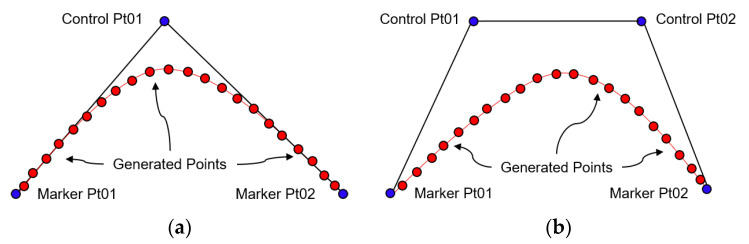
Functions for smoothing polygon curve. (**a**) Quadratic Bezier; (**b**) cubic Bezier.

The original marked points from the previous steps are not enough on their own to form a smoother shape of the polygon. Hence, the Bezier Curve was utilized to generate additional points between the marked points to show a much smoother polygon. But using the Bezier curve requires the use of control point(s) to trace the path of the function. We narrowed the choice between Quad Bezier (Figure 5a) or the Cubic Bezier (Figure 5b).

The Quadratic Bezier curve mathematically represented in Equation (1) is a path that is traced by the function *B_q_*(*t*), given the points *M*_1_, *M*_2_, and *C*_1_.

(1) $$\begin{array}{c} B_{q}\left(t\right)=\left(1-t\right)\left[\left(1-t\right)\left(M_{1}\right)+t\left(C_{1}\right)\right]+t\left[\left(1-t\right)\left(C_{1}\right)+t\left(M_{2}\right)\right],0\leq t\leq 1\\ ={\left(1-t\right)}^{2}M_{1}+2\left(1-t\right)tC_{1}+t^{2}M_{2},0\leq t\leq 1 \end{array}$$

where *M*_1_ is the first marked point, *M*_2_ is the succeeding marked point, and *C*_1_ is the control point. As *t* changes value from 0 to 1, the curve leaves *M*_1_ into the direction of *C*_1_, and then the curve bends to finally arrive at *M*_2_ from the direction of *C*.

For the cubic Bezier curve (Equation (2)), it is a path traced by the function *B_c_*(*t*), given the points *M*_1_, *M*_2_, *C*_1_, and *C*_2_.

(2) $$B_{c}\left(t\right)={\left(1-t\right)}^{3}M_{1}+3{\left(1-t\right)}^{2}tC_{1}+3\left(1-t\right)t^{2}C_{2}+t^{3}M_{2},0\leq t\leq 1$$

where *M*_1_ and *M*_2_ are the first and succeeding marked points, respectively, while *C*_1_ and *C*_2_ are the first and second control points. As time *t* increases, the curve starts from point *M*_1_ going toward the direction of the control points *C*_1_ and *C*_2_, but then it bends to finish at the point *M*_2_. The cubic Bezier curve usually will not pass through the control points *C*_1_ and *C*_2_, since these points are only used for directional information. The distance between the control points determine the speed and distance that the curve moves into the direction of *C*_1_ before it turns toward *C*_2_.

**Figure 6:**
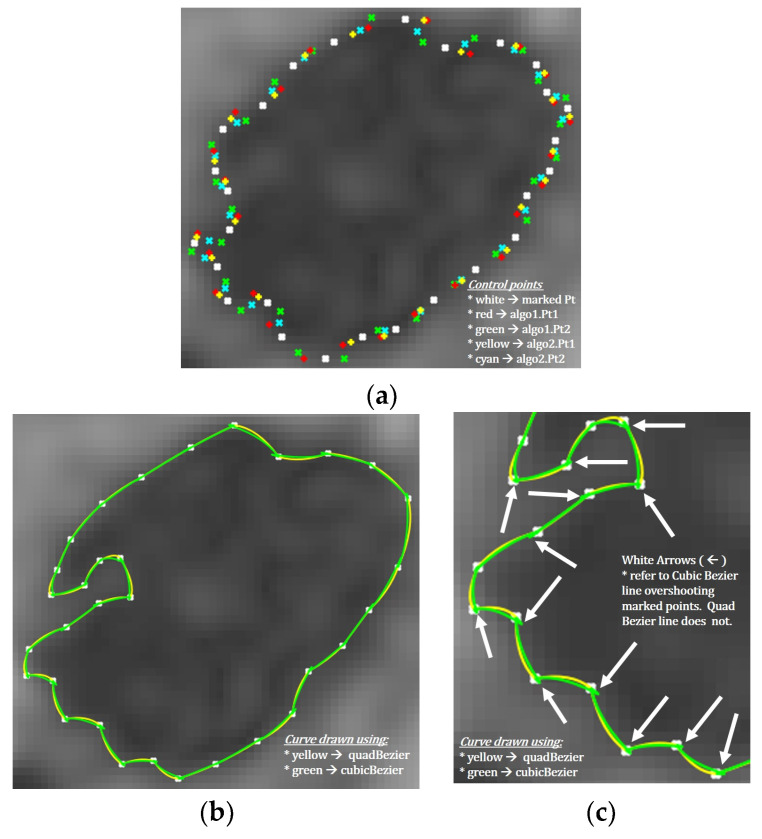
Quad- and curve-Bezier curves. (**a**) Control and generated points; (**b**) traced path; (**c**) cubic-Bezier overshooting.

The cubic Bezier function *B_c_*(*t*) could be described as the affine combination of two quadratic Bezier functions, similar to Equation (1), represented in Equation (3).

(3) $$B_{c}\left(t\right)=\left(1-t\right)B_{M_{1},C_{1},C_{2}}\left(t\right)+tB_{C_{1},C_{2},M_{2}}\left(t\right),0\leq t\leq 1$$

where *B*_*M*1,*C*1,*C*2_(*t*) is the quadratic Bezier curve function for marked point $M_{1},$ control points *C*_1_ and *C*_2_. On the other hand, the quadratic Bezier function *B_C_*_1,*C*2,*M*2_(*t*) is for the control points *C*_1_, *C*_2_ and marked point *M*_2_. Visually comparing the results between quad Bezier and cubic Bezier (Figure 6), quadratic Bezier gave the smoothest curve without overshooting the marked points. Hence quadratic Bezier curve function was chosen for this purpose.

#### 4.4.2. Generation of the 3D View

**Figure 7:**
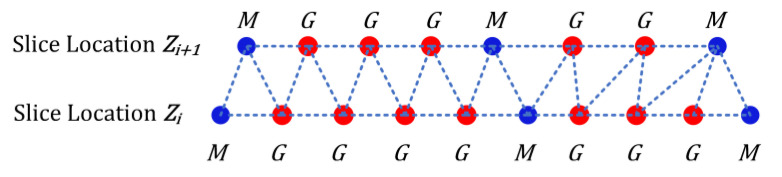
OpenGL Triangle forming using marked and generated points.

This part of the module depends heavily on the OpenGL libraries since it involves generating a 3D rendering of the lumen and wall (depending on the doubly-linked list passed from the marking module steps). The marking points are extracted from the doubly linked list and converted to polygons (triangles to be exact) between the succeeding layers, which are subsequently filled with texture patterns for better viewing (Figure 7).

The marked plus generated points that form the corners of the triangle, known as vertices, are insufficient on their own to generate the openGL triangle; the texture coordinates and vertex normal attributes also need to be passed to it in order to be saved as the vertex, texture, and normal buffers of the triangle generated. These vertex points are then projected onto the space with the corresponding scaling and rotations in order to display the three-dimensional object in the necessary view, depending on the values set for the projection, view, and model matrices.

## 5. Resulting System

**Figure 8:**
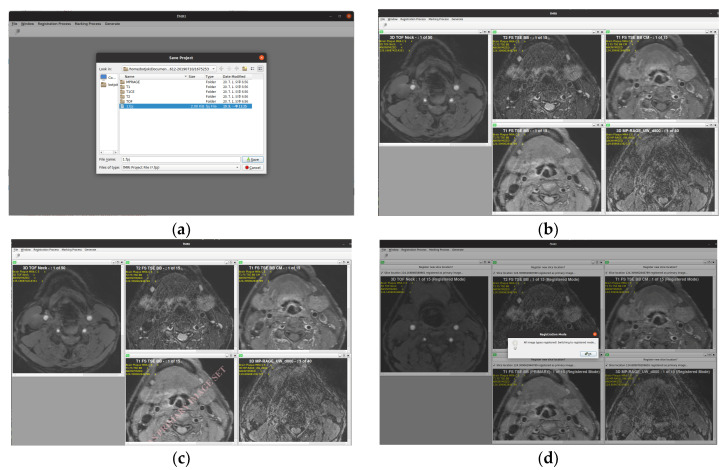

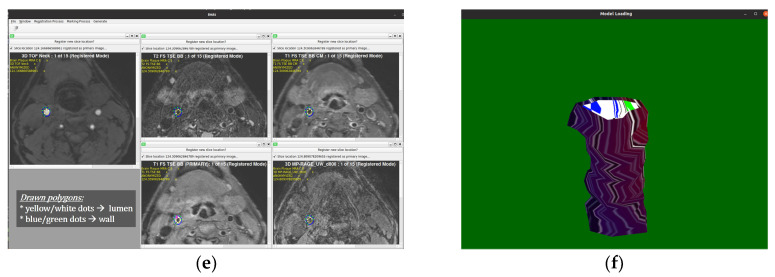
The MRI-CAPIDS. (**a**) Browsing DICOM files; (**b**) initial loading; (**c**) primary set selection; (**d**) registration; (**e**) marked images; (**f**) generated 3D rendering.

The design and development process is conducted using the RBCDWBPA methodology (Rapid By-Customer Demand with Business Process Approach) in conjunction with SFA (Systems Features Analysis). In the developed system, regardless of the name given to the sub-directory folder (Figure 8a), the classification type utilized for the process is extracted from the DICOM file itself, not based on the name of the sub-directory folder. Additional features included in the developed system are the ability to zoom in or out as well as panning the view horizontally or vertically. Scrolling, zooming, and panning taking place on a particular MRI-type window are simultaneously performed on the five other types. 

The current version displays several forms of tag data for basic information purposes only: Series Description (Image type), Study Description, and Patient Name (instead displayed as Anonymous) (Figure 8b). After the primary image set has been selected (Figure 8c), the loaded DICOM files are then registered (Figure 8d). The registered image sets are then ready for marking of lumen and walls (Figure 8e), with the markings then being utilized to generate the 3D-rendered image (Figure 8f).

## 6. Conclusions

This study has presented the use of entirely open-source tools in the design and development of a highly specific software system to assist medical practitioners in processing carotid MRI images, as well as for providing patients with a better visualization of their condition. The initial version of the developed system represents a proof-of-concept that is open-source, platform-independent, and can be expanded to accommodate either a few or a selected number of additional features, to avoid both of feature-bloat and feature-creep. The current version of the program is classified as a research-only tool for now up until a version submitted to relevant authorities, such as the Ministry of Food and Drug Safety, can be approved for clinical/medical use in the future.

There was a minimum of 10,000% improvement in the image loading and display of the utilized QDCM library while the quadratic Bezier function was used to smoothen the curve of the polygon formed by the marking points. It was also designed to be as simple as possible to use, with only minimal procedural steps. A limitation of our work is the manual processes in the system especially the process manually marking and identifying the wall and lumen. This will be taken into consideration for future works wherein the first step would be utilizing image processing and then explore the possibility of using AI/deep learning with the review paper of Jodas et al. [39] being a good place to start. It is envisioned that this work would be further improved to include the possibility of being integrated into existing or new electronic health records (EHR) which have been shown to help expand health services offered to people especially in remote locations [40].

## Figures and Tables

**Table 1:** Conventional and modified American Heart Association (AHA) classification of atherosclerotic plaque.

Conventional AHA Classification (Stary)	Modified AHA Classification for MRI (Cai et al. [8])
Type I: initial lesion with foam cells	Type I–II: near-normal wall thickness, no calcification
Type II: fatty streak with multiple foam cell layers	
Type III: preatheroma with extracellular lipid pools	Type III: diffuse intimal thickening or small eccentric plaque with no calcification
Type IV: atheroma with a confluent extracellular lipid core	Type IV–V: plaque with a lipid or necrotic core surrounded by fibrous tissue with possible calcification
Type V: fibroatheroma	
Type VI: complex plaque with possible surface defect, hemorrhage, or thrombus	Type VI: complex plaque with possible surface defect, hemorrhage, or thrombus
Type VII: calcified plaque	Type VII: calcified plaque
Type VIII: fibrotic plaque without lipid core	Type VIII: fibrotic plaque without lipid core and with possible small calcifications

**Table 2:** Improvement in the conversion to QImage for display.

Image to QImage Conversion Algorithm	MRI Types				
	3D MP-RAGE_UW_d8000	T1 FS TSE BB	T1 FS TSE BB CM	T2 FS TSE BB	3D TOF Neck
Original QDCM	74.00 ms	249.67 ms	249 ms	244.33 ms	280.33 ms
Modified	0.67 ms	2.33 ms	1.67 ms	2.00 ms	1.67 ms
Factor Improvement	11,100.00%	10,700.00%	14,940.00%	12,216.67%	16,820.00%
